# Caregiver Challenges and Functional Requirements for an mHealth App Supporting Home-Based Dementia Care: Qualitative Interview Study

**DOI:** 10.2196/87234

**Published:** 2026-09-16

**Authors:** Muhammad Fariz Eda Andhika, Naiya Dwita Ayunir, Raudhatul Jannah, Putu Wuri Handayani, Kamila Alifia Imanuddin

**Affiliations:** 1 Faculty of Computer Science University of Indonesia Depok, West Java Indonesia

**Keywords:** mobile health, mHealth, digital solution, dementia, caregiver, Indonesia

## Abstract

**Background:**

The growth of the older adult population in Indonesia since 2019 has contributed to a 2.46% increase in dementia cases, with the number of cases projected to increase 2.5-fold by 2050. This trend is exerting a major impact on older adults and increasing the burden on caregivers. The current lack of systemic health support suggests that digital solutions—such as mobile health (mHealth) apps—have the potential to support caregivers and people living with dementia. However, to date, no mHealth initiatives have been developed through a participatory approach that involves users in the design process in accordance with the sociocultural conditions in Indonesia.

**Objective:**

This study aimed to identify the challenges faced by dementia caregivers in home care settings and to determine the functional requirements for an mHealth app to support them.

**Methods:**

The study adopted a qualitative participatory design, interviewing 12 health care workers, 2 employees of nongovernmental organizations, and 35 dementia caregivers. The qualitative data were analyzed using content analysis. The challenges identified by the interviewees were mapped onto the app design process as functional requirements.

**Results:**

The challenges identified in this study include a lack of education and awareness regarding the symptoms and treatment of dementia, the stress and mental strain experienced by caregivers, difficulties in monitoring the health and daily lives of people with dementia, barriers to access to health services, and declining cognitive function among older adults with dementia. To overcome these challenges, we identified corresponding functional requirements for the mHealth app, including diaries, music playlists, community forums, and quizzes and games for people with dementia, as well as dementia education videos and psychological assessments for caregivers. Aspects such as ease of use and the quality of information influenced the acceptance of the app by people with dementia and their caregivers. Educational materials, such as short videos, articles, and infographics, addressed dementia symptoms and steps that can be taken at home to care for people with dementia. Psychological assessments were developed using a validated tool. Quizzes and games helped caregivers design fun, relevant questions based on the personal experiences of adults with dementia. Music playlists highlighted songs that suited the mood of people with dementia, enhancing their personalized experiences without needing explicit guidance from the app. Community forums encouraged caregivers to voluntarily share their experiences and support others, strengthening their sense of belonging and emotional involvement in the caregiving process. Diaries could be used by caregivers to record the daily condition of people with dementia and their care.

**Conclusions:**

The challenges and functional requirements identified in this study provide a practical foundation for organizations and facilities to develop culturally appropriate health apps for dementia home care in Indonesia.

## Introduction

The global population aged ≥60 years is increasing, with an estimated rise of around 400 million since 2020 [1], and it is expected to double by 2050, reaching 2.1 billion [1]. In Indonesia, according to data from the Central Statistics Agency [2], this trend in the older adult population, which began in 2021, is marked by 11.75% of the Indonesian population being classified as older adults. This increase is not only reshaping the country’s demographic structure but also poses new challenges in the health sector, especially in relation to neurodegenerative diseases such as dementia [2]. One of the most crucial issues is the increasing prevalence of neurodegenerative diseases (eg, dementia, Parkinson disease, amyotrophic lateral sclerosis, and Alzheimer disease), which are progressive disorders that affect the central nervous system and cause the death of nerve cells [3]. These diseases affect cognitive function and the motor, sensory, and emotional systems [3].

In 2019, 26 million new cases of neurological diseases were recorded in Indonesia [4]. Alzheimer disease and other forms of dementia accounted for 5.46% of the total cases, which are projected to increase 2.5-fold by 2050 [4]. Indonesia is experiencing a surge in dementia prevalence, with a projection from around 9 per 1000 people in 2021 to >25 per 1000 by 2050, giving it one of the highest prevalence growth rates in Southeast Asia [5]. Of all types of neurodegenerative diseases, dementia is one of the most common conditions that progressively affects cognitive function [6]. Therefore, there is a need for digital innovations—such as mobile health (mHealth) apps—that make it possible to manage this disease in a more integrated and sustainable manner [7]. mHealth helps people with dementia carry out daily activities such as remembering schedules, performing cognitive exercises, and managing medications independently [8]. mHealth also supports people with dementia in staying socially active through features such as reminiscence therapy and discussion forums [9].

In addition to medical treatment, nonpharmacological approaches, such as daily care, emotional support, and assistance with daily living activities, are crucial in managing dementia [10]. In most cases in Indonesia, families are the main party responsible for dementia care, making them a key element in the success of the treatment process and the improvement of patients’ quality of life [11]. The presence of the family as primary caregivers has been proven to provide a sense of security and comfort for people with dementia [11]. In addition to providing emotional closeness, families that provide knowledge and support in social, spiritual, and economic aspects play an important role in maintaining stability for older adults [11]. However, families often face obstacles in this context, such as a lack of knowledge about the illness, physical and mental exhaustion, and difficulties in managing neuropsychiatric symptoms experienced by older adults, such as delusions, aggressive behavior, and sleep disorders [11].

Research on mHealth for dementia care remains limited, with existing studies focusing on mHealth acceptance in Indonesia [7] and dementia app design in Australia [12]. Moreover, people living with dementia often have difficulty using technology in their daily lives, and dementia caregivers need knowledge about the treatments and symptoms of dementia and about managing people with dementia [7]. Therefore, this study addresses the following research question: What are the challenges faced by dementia caregivers in home care settings, and what functional requirements should an mHealth app address to support them?

This study provides guidance for health technology developers and health facilities to develop apps for dementia home care.

## Methods

### Study Design and Population

This study adopted a qualitative approach, collecting data through in-depth interviews. The COREQ (Consolidated Criteria for Reporting Qualitative Research) guidelines were used as a comprehensive checklist to ensure complete reporting of the key components of qualitative research (Multimedia Appendix 1) [13]. We conducted interviews with 12 experts (physicians and psychologists), 2 employees of nongovernmental organizations (NGOs), and 35 dementia caregivers. The respondents were aged between 25 and 50 years and resided on the island of Java. Snowball sampling was used to recruit dementia caregivers. Interviews were conducted with NGO employees because they had extensive knowledge of and experience with dementia, with expertise in subjects such as stigma, access to services, and the needs of dementia caregivers. Interviews with experts were conducted to determine the medical and scientific aspects most relevant to the problems in dementia treatment. Dementia caregivers were also consulted regarding the identification of challenges because they had firsthand experience caring for people with dementia. The interviews focused on identifying the challenges experienced by each party and the corresponding functional requirements for the dementia home care app. We provided a brief explanation of the study objectives before conducting the interviews. Each interview lasted for 45 to 60 minutes and was conducted either face-to-face or online. We recorded and transcribed the interviews, ensuring that all data were anonymized.

Multimedia Appendix 2 presents the questions addressed to the experts, which focused on professional backgrounds, challenges in diagnosis, and methods for treating patients with dementia. The interviews with NGO representatives aimed to understand their role in managing dementia, the challenges they have faced, and the programs they have developed (Multimedia Appendix 3). The questions for dementia caregivers focused on their firsthand experience of caring for people with dementia, including the challenges of caring for these individuals, strategies for communicating with these individuals and health workers, and the support they need (Multimedia Appendix 4).

### Analysis Methods

Content analysis is a qualitative approach used to interpret the meaning of textual data through systematic classification, categorization, and pattern identification [14]. This technique is suitable for analyzing data from interviews, observations, or documentation, especially when the research focus is on exploring perceptions, meanings, and subjective experiences [14]. According to Bengtsson [15], the content analysis process generally begins with the researchers familiarizing themselves with the data through repeated reading, followed by open coding or labeling of important units of meaning. The derived codes are then grouped into categories based on their similarities and relationships. The primary theme is then deduced to answer the research question. In this case, after the interviews, we discussed the responses to determine the point of data saturation. The first, second, and third authors coded the data, and all authors held meetings to discuss the coding process.

The content analysis was carried out to identify problems raised by each category of interviewees (health workers and caregivers). The collected data were grouped based on important commonalities such as challenges in caring for people with dementia and expected caregiver needs. The results of this content analysis provided the basis for designing an app that addresses user needs.

### Ethical Considerations

All respondents provided written consent to participate in this study, and all respondent data were anonymized. This study was approved by the ethics committee of the Faculty of Computer Science, University of Indonesia (KEPS4-1/072026).

## Results

### Challenges Faced by Caregivers and People With Dementia

In Indonesia, people with dementia face a variety of complex social, emotional, and cognitive challenges. The Ministry of Health of the Republic of Indonesia [16] explains that dementia causes a decline in functions such as memory, behavior, and cognitive ability, which impacts people’s ability to carry out daily activities independently. People with dementia also experience personality changes, agitation, paranoia, and total dependence on caregivers in the advanced stages of the disease [16]. The inability to maintain personal hygiene can even increase the risk of infection and malnutrition [16]. People with dementia often make important decisions related to finances, personal safety, and social relationships that can negatively impact their well-being and that of their families [10].

Multitasking difficulties and decreased cognitive flexibility are further significant challenges [17]. Older adults often have difficulty completing tasks that require several steps, such as preparing food or using household appliances, due to limited memory and attention [17]. They often face confusion when connecting objects to their functions, so adapting to the environment becomes a challenge in itself [17].

Sleep problems are another challenge related to dementia. People with dementia tend to experience circadian rhythm disturbances characterized by difficulty sleeping at night and excessive sleepiness during the day, which affect health and trigger behavioral symptoms such as agitation or aggression [18,19]. Emotional impacts, such as depression and anxiety, cannot be ignored, as feelings of helplessness and loss of control over life are often triggers for labile moods [18]. These emotional and psychological burdens create challenges for dementia caregivers, who often experience prolonged stress, fatigue, and even depression due to increasing responsibilities [19]. This stress is further exacerbated when people with dementia experience behavioral and psychological symptoms that are difficult to control, such as agitation, aggression, and hallucinations [19].

Dementia caregivers can also experience emotional conflict when personality changes make the people they care for seem unrecognizable compared to who they were before the onset of the disease [20]. Many dementia caregivers experience social isolation due to limited time and energy to interact with the surrounding environment [21]. This stems from the high demands of their care duties, which often make it difficult for them to engage in social activities or seek support from their communities. Consequently, they lose access to social support systems that can help them manage the stress and emotional burdens that arise from their caregiving responsibilities [21].

As Multimedia Appendix 5 shows, the main challenges identified in the content analysis include a lack of education and awareness regarding the symptoms and treatment of dementia, stress and mental strain experienced by caregivers, difficulties in monitoring the health and daily lives of people with dementia, barriers impeding access to health services, and declining cognitive function among people with dementia.

The evidence suggests that dementia home care apps should be designed primarily for use by caregivers who assist people with dementia. The challenges derived from the interviews are consistent with those reported in research by Rettinger et al [22], Gonzalez-Gomez et al [23], Dinakrisma et al [24], Ye et al [25], Zou et al [26], and Nasrun et al [7]. Accordingly, the features and interface of the app must be arranged so that the app can be easily used by dementia caregivers, either independently or in the presence of people with dementia, while still prioritizing aspects such as accessibility, ease of use, and suitability for the cognitive limitations of older users.

### Functional Requirements for Dementia Home Care Apps

The intervention theory developed by Argyris [27] was used as a foundation for designing a system that is not only informative but also capable of empowering users to make independent and reflective decisions. The use of this theory is important because the main problems faced by dementia caregivers and people with dementia extend beyond limited information and include low confidence, control, and commitment to carrying out the treatment process consistently [28]. Moreover, intervention theory offers a framework for explaining how an intervention can positively affect user behavior through 3 main principles: the provision of valid and useful information, the freedom for the user to act based on the information obtained (free informed choice), and the formation of an internal commitment to the chosen action (internal commitment) [28]. Thus, intervention theory helps bridge users’ cognitive, affective, and behavioral dimensions, which is particularly relevant in the context of dementia care, which is fraught with psychosocial challenges and informal decision-making at the individual level.

Table 1 presents the required features based on challenges identified by Rettinger et al [22], Gonzalez-Gomez et al [23], Dinakrisma et al [24], Ye et al [25], Zou et al [26], and Nasrun et al [7]. Table 1 shows that the most important features are dementia education, medical management (ie, reminders for medication and appointments), condition tracking, and relaxation. Other features that are also important for people with dementia and their caregivers include early detection, caregiver forums, teleconsultations, and cognitive stimulation. These results suggest that the development of digital solutions for people with dementia and their caregivers should prioritize features identified by researchers while also accounting for less frequently noted features to optimize the app’s effectiveness and sustainability.

**Table 1:** Research-based mapping of challenges to app features.

Challenges and app features			Rettinger et al [22]		Gonzalez-Gomez et al [23]		Dinakrisma et al [24]		Ye et al [25]		Zou et al [26]		Nasrun et al [7]
**Lack of education and awareness regarding dementia symptoms and management**													
	Dementia education (symptoms, therapy, and treatment)	✓		✓		✓				✓		✓	
	Early detection (symptoms and causes of dementia)	✓										✓	
**Caregivers experiencing stress and mental strain while caring for people with dementia**													
	Caregiver forum (sharing experiences and support)			✓						✓			
	Relaxation (exercises and stress-reduction techniques)	✓		✓		✓		✓					
**Difficulties in monitoring the health and daily lives of people with dementia**													
	Medical management (reminders for medication and appointments)			✓		✓		✓		✓		✓	
	Condition tracking (mood tracker, symptom checks, and behavior changes)	✓		✓		✓				✓		✓	
Barriers impeding access to health services: teleconsultation feature							✓				✓		✓
Declining cognitive function among people with dementia: cognitive stimulation feature			✓						✓				✓

We next mapped the required features based on the results of the literature review and content analysis onto the principles of intervention theory to ensure that the app’s features were not only functionally relevant but also consistent with theoretical guidance. Multimedia Appendix 6 describes this mapping of features onto the principles of intervention theory.

The features defined in Multimedia Appendix 6 were grouped into 4 categories of health service approaches: promotive, preventive, curative, and rehabilitative. The promotive approach focuses on increasing user knowledge, awareness, and engagement in proactively maintaining health. Preventive methods aim to prevent or detect potential disturbances early through periodic monitoring and assessment. The curative approach supports the ongoing process of treating the condition through access to medical services and routine monitoring. Meanwhile, the rehabilitative dimension is geared toward helping to restore the user’s cognitive, emotional, and social functions through activities that are therapeutic and enjoyable.

Dementia education features, dementia caregiver psychological assessments, and community forums are designed to support the promotive and preventive aspects by increasing literacy, awareness, and social support for dementia issues. Meanwhile, app features such as daily logs and online consultations are aimed at facilitating the role of caregivers in informal curative and rehabilitative care, although they do not yet include direct clinical interventions. Psychosocial support is also enhanced through community forums, addressing needs that are not addressed in Wilson et al [29]. Features such as diaries and community forums were designed based on dementia caregivers’ expectations for caring for people with dementia. The app’s interactive educational features, comprising short videos, articles, and infographics, were also seen as useful, especially by health workers, for explaining dementia to the families of affected people in easy-to-understand language.

## Discussion

### Principal Findings

The challenges faced by people with dementia and their caregivers include a lack of education and awareness regarding dementia symptoms and treatment, caregiver stress and mental strain, difficulties in monitoring the health and daily lives of people with dementia, barriers to accessing health care services, and declining cognitive function among people with dementia. The corresponding functional requirements for an mHealth app include dementia education, caregiver psychological assessments, quizzes and games, music playlists, community forums, and diaries.

These functional requirements were mapped onto the necessary design principles and feature requirements. The first design principle is user-centered readability and accessibility, which ensures that the app is easy to use and read for older users, who generally have physical and cognitive limitations. The implementation includes the use of large and clear fonts, simple icons, and intuitive navigation that does not confuse users. This approach helps reduce user barriers to understanding the content of the app and navigating it.

The second design principle is contextual and actionable information delivery, which involves providing relevant information according to the user’s situation that can be immediately acted on. This principle was implemented with features such as diaries, which help caregivers provide daily care for older people.

The third design principle is emotional and personal engagement, which aims to increase users’ emotional engagement through personalization. This principle was implemented through a personal memory-based quiz feature to stimulate the memories of older people. This feature not only stimulates memory but also enhances the user’s emotional connection to the app.

The fourth principle concerns the empowerment of user autonomy, which gives users the opportunity to make independent decisions about the use of the app. The implementation includes options for users to choose daily activities and types of educational content. This increases users’ sense of control over their app experience.

The final principle is validation-based iterative refinement, which emphasizes the importance of refining the design based on continuous evaluation by users. This principle will be applied by conducting regular usability evaluations to ensure that the app is continually updated in a manner that responds to user needs.

### Theoretical and Practical Implications

Rathnayake et al [12], Wilson et al [29], and Engelsma et al [30] and have demonstrated the importance of inclusive and user-based apps in supporting people with dementia and their caregivers. However, Nasrun et al [7] specifically noted that the development of mHealth in Indonesia still faces obstacles in terms of adapting to the local culture, limited access, and a lack of understanding of caregiver needs. This study contributes to overcoming the challenges faced by people with dementia and caregivers by making recommendations for functional solutions needed in dementia home care apps. The required functional features were also mapped onto the principles of intervention theory.

Health facilities, such as hospitals, geriatric clinics, and health centers, can use the app’s features to monitor people with dementia, allowing health workers to access medication adherence data, scores from cognitive exercises, and daily activities recorded by families. Educational materials in the form of short videos, articles, and infographics related to dementia are also needed by people with dementia and their caregivers so that they can improve their knowledge and skills for managing dementia.

For dementia caregivers and families, apps can help record daily routines such as taking medication, performing activities, and keeping notes or photos as reminders or references. All logging is performed in 1 app, so there is no need to take notes manually. The discussion forum in the app also allows fellow dementia caregivers to share experiences and seek advice. The educational materials can help users understand the symptoms of dementia and the steps that can be taken at home.

For governments and policymakers, this app can be used to collect anonymous data on the number of dementia cases, their spread, and the patterns of activity among affected individuals. This information can provide the basis for developing caregiver training programs, developing home care services, or allocating health budgets for dementia treatment. The government can encourage app distribution through community service facilities, community health posts (*posyandu*), and digital communities. Basic digital training for people with dementia and their caregivers can be offered as a companion program to increase the success of the app’s implementation.

For the public, apps can help reduce the stigma around dementia by providing easily accessible information about symptoms, treatment methods, and support. Through notifications or in-app campaigns, information about dementia can be disseminated to the wider community. Collaboration with dementia organizations and communities can also expand the reach of information dissemination, especially in areas lacking access to dementia-related education and services.

### Limitations

This study did not involve people with dementia because the condition inherently impedes full participation in this study, so dementia caregivers served as the main respondents. In addition, the average respondent involved in this study was familiar with technology, which may have influenced the results of the evaluation, which do not necessarily reflect the experiences of users from areas with low levels of digital literacy. The respondents also resided on the island of Java. Thus, future research could involve people with dementia, users with lower digital literacy, and those in rural areas. Future research could also implement home care apps for dementia and evaluate their implementation.

### Conclusions

This study found that mHealth apps for dementia care can be designed in an inclusive and user-oriented manner to address the challenges associated with dementia care. The main challenges faced by dementia caregivers are a lack of education and awareness regarding the symptoms and treatment of dementia, the stress and mental strain experienced by dementia caregivers, difficulties in monitoring the health and daily lives of people with dementia, barriers impeding access to health services, and declining cognitive function among people with dementia. We also identified the need for functional features aimed at supporting dementia caregivers and experts in caring for people with dementia, namely educational features, psychological assessments, community forums, diaries, quizzes and games, and music playlists. These features are designed to complement each other in creating a friendly digital environment, supporting caregiving activities, and paying attention to the cognitive and emotional aspects of users.

Further research is expected to continue until the app implementation stage so that its functionality can be tested directly with users to determine its effectiveness and acceptance. Further research is suggested to encourage the use of supporting technologies, such as wearable devices and health sensors, to provide additional support for users with cognitive limitations.

## Appendix

Multimedia Appendix 1 COREQ (Consolidated Criteria for Reporting Qualitative Research) checklist.

Multimedia Appendix 2 Interview questions for health workers.

Multimedia Appendix 3 Interview questions for nongovernmental organization employees.

Multimedia Appendix 4 Interview questions for dementia caregivers.

Multimedia Appendix 5 Summary of challenges identified through content analysis.

Multimedia Appendix 6 Mapping of challenges, features, principles of intervention theory, and health efforts.

## Abbreviations

COREQ: Consolidated Criteria for Reporting Qualitative Research
mHealth: mobile health
NGO: nongovernmental organization

## Data Availability

The datasets generated or analyzed during this study are available from the corresponding author on reasonable request.
