# Supplementary material for: Caregiver Challenges and Functional Requirements for an mHealth App Supporting Home-Based Dementia Care: Qualitative Interview Study
Source: JMIR Form Res. 2026 Sep 16;10:e87234. doi: 10.2196/87234 (PMC13628036; doi:10.2196/87234)
Supplement: Supplementary file 1 [file formative_v10i1e87234_app1.docx]

## Multimedia Appendix 1 Consolidated Criteria for Reporting Qualitative Studies (COREQ) checklist

| **No. Item** | **Guide questions/description** | **Reported on Manuscripts** |
| --- | --- | --- |
| **Domain 1: Research team and reﬂexivity** | | |
| *Personal Characteristics* | | |
| 1. Interviewer/ facilitator | Which author/s conducted the interview or focus group? | First, second and third author |
| 2. Credentials | What were the researcher’s credentials? E.g., PhD, MD | Bachelor students |
| 3. Occupation | What was their occupation at the time of the study? | First, second and third author: Undergraduate students; fourth and fifth authors: lecturers |
| 4. Gender | Was the researcher male or female? | First author: male; second until fifth author: female |
| 5. Experience and training | What experience or training did the researcher have? | N/A |
| *Relationship with participants* | | |
| 6. Relationship established | Was a relationship established prior to study commencement? | No prior relationships with respondents |
| 7. Participant knowledge of the interviewer | What did the participants know about the researcher? e.g., personal goals, reasons for doing the research | Researchers provided a brief explanation of the study objective before conducting the interview |
| 8. Interviewer characteristics | What characteristics were reported about the interviewer/facilitator? e.g., Bias, assumptions, reasons and interests in the research topic | N/A |
| **Domain 2: study design** | | |
| *Theoretical framework* | | |
| 9. Methodological orientation and theory | What methodological orientation was stated to underpin the study? e.g., grounded theory, discourse analysis, ethnography, phenomenology, content analysis | Content analysis in the analysis method section |
| *Participant selection* | | |
| 10. Sampling | How were participants selected? e.g., purposive, convenience, consecutive, snowball | Snowball sampling in the study design and population section |
| 11. Method of approach | How were participants approached? e.g., face-to-face, telephone, mail, email | Face-to-face or online meeting in the study design section |
| 12. Sample size | How many participants were in the study? | 12 experts (doctors and psychologists), two non-governmental organizations (NGOs) employees, and 35 dementia caregivers in the study design section |
| 13. Non-participation | How many people refused to participate or dropped out? Reasons? | N/A |
| *Setting* | | |
| 14. Setting of data collection | Where was the data collected? e.g., home, clinic, workplace | Online meetings using phone, Zoom Meetings and/or workplace in the NGO office |
| 15. Presence of non-participants | Was anyone else present besides the participants and researchers? | N/A |
| 16. Description of sample | What are the important characteristics of the sample? e.g., demographic data, date | The aged respondents between 25 until 50 years old and the domicile of respondents in the Java island. |
| *Data collection* | | |
| 17. Interview guide | Were questions, prompts, guides provided by the authors? Was it pilot tested? | The interview questions are provided in Multimedia Appendix 2,3,4. No pilot testing. |
| 18. Repeat interviews | Were repeat interviews carried out? If yes, how many? | N/A |
| 19. Audio/visual recording | Did the research use audio or visual recording to collect the data? | Interviews were audio-recorded |
| 20. Field notes | Were ﬁeld notes made during and/or after the interview or focus group? | N/A |
| 21. Duration | What was the duration of the interviews or focus group? | 45-60 minutes |
| 22. Data saturation | Was data saturation discussed? | The authors discussed with each other after the interview and decided when data saturation occurred |
| 23. Transcripts returned | Were transcripts returned to participants for comment and/or correction? | N/A |
| **Domain 3: analysis and ﬁndings** | | |
| *Data analysis* | | |
| 24. Number of data coders | How many data coders coded the data? | The first, second and third authors coded the data and the authors also held meetings to discuss the coding process |
| 25. Description of the coding tree | Did authors provide a description of the coding tree? | Table 1 |
| 26. Derivation of themes | Were themes identiﬁed in advance or derived from the data? | Themes were derived from the data |
| 27. Software | What software, if applicable, was used to manage the data? | N/A |
| 28. Participant checking | Did participants provide feedback on the ﬁndings? | N/A |
| *Reporting* | | |
| 29. Quotations presented | Were participant quotations presented to illustrate the themes/ﬁndings? Was each quotation identiﬁed? e.g., participant number | Table 1 |
| 30. Data and ﬁndings consistent | Was there consistency between the data presented and the ﬁndings? | Yes |
| 31. Clarity of major themes | Were major themes clearly presented in the ﬁndings? | Table 1 |
| 32. Clarity of minor themes | Is there a description of diverse cases or discussion of minor themes? | N/A |
