# Supplementary material for: Caregiver Challenges and Functional Requirements for an mHealth App Supporting Home-Based Dementia Care: Qualitative Interview Study
Source: JMIR Form Res. 2026 Sep 16;10:e87234. doi: 10.2196/87234 (PMC13628036; doi:10.2196/87234)
Supplement: Supplementary file 2 [file formative_v10i1e87234_app2.docx]

# Multimedia Appendix 2. Interview Questions for Health Workers

| **Category** | **Questions** |
| --- | --- |
| Demographic questions | Provide brief information about yourself (age, domicile) |
|  | Can you describe your professional or educational background? |
|  | What are your main specialties or interests in the medical field (e.g., neurology, geriatrics)? |
| Challenges faced by doctors | What are some of the key challenges you see in dementia diagnosis and treatment today? |
| Challenges faced by people with dementia | What are the symptoms that most often make people with dementia feel difficult in daily activities? |
|  | To what extent do people with dementia typically understand their disease condition, and how does this affect their disease management? |
| Challenges in care faced by caregivers | How do families or caregivers usually communicate with doctors regarding the development of people with dementia? Are there any obstacles? |
|  | What are some skills or knowledge that caregivers often lack in taking good care of people with dementia? |
| The needs of people with dementia and caregivers | What facilities or services do people with dementia need most to improve their quality of life? |
|  | What do you think are the main needs of people with dementia and their family in supporting daily care? What support is needed/can be provided related to these needs? |
|  | Can you prioritize support into essential, nice to have, and nonessential categories? |
|  | If a digital solution is going to be designed to help people with dementia and caregivers, what do you think is the top priority that should be met? |
| Design aspects from a medical perspective | Are there any aspects of app design that you think need to be addressed to meet the needs of users in the context of apps for dementia treatment? |
| The role of technology from the medical side | What are your expectations for the use of technology in improving the quality of life of people with dementia? |
|  | How do you see the role of technology in addressing the issues faced by people with dementia? |
