# Supplementary material for: Caregiver Challenges and Functional Requirements for an mHealth App Supporting Home-Based Dementia Care: Qualitative Interview Study
Source: JMIR Form Res. 2026 Sep 16;10:e87234. doi: 10.2196/87234 (PMC13628036; doi:10.2196/87234)
Supplement: Supplementary file 3 [file formative_v10i1e87234_app3.docx]

# Multimedia Appendix 3. Interview Questions for NGO Employees

| **Category** | **Question** |
| --- | --- |
| Support for caregivers and people with dementia | How does your organization support people with dementia and caregivers in addressing the challenges they face on a daily basis? |
|  | What are the main challenges your organization faces in providing emotional support and education to people with dementia and caregivers? |
|  | How does your organization involve caregivers in the process of developing a program or service to ensure their needs are truly met? |
| Education and public awareness | What strategies is your organization implementing to increase public awareness of dementia and the importance of supporting caregivers? |
|  | How does your organization ensure that education about dementia reaches all walks of life, including those with limited access to resources? |
|  | What is the biggest challenge your organization faces in delivering dementia education to the wider community, and how can you overcome it? |
| Technology collaboration and implementation | Has your organization ever worked with technology developers to support treatment of people with dementia? If so, what is the process? If not, how necessary is the implementation of technology for supporting treatment of people with dementia and helping caregivers caring for people with dementia to prosper? |
|  | What is your organization’s view on the use of mHealth in helping caregivers and people with dementia in Indonesia? Is mHealth for dementia needed in Indonesia? |
| Social and community support | How does your organization see the role of the community in supporting caregivers of people with dementia, and what can be done to strengthen that role? |
| Caregiver experience | According to your organization, what are the most common experiences or challenges that caregivers of people with dementia face when managing their daily lives with people with dementia? |
|  | What is the best way for caregivers to receive emotional support and education in caring for people with dementia, according to your organization? |
