# Supplementary material for: Caregiver Challenges and Functional Requirements for an mHealth App Supporting Home-Based Dementia Care: Qualitative Interview Study
Source: JMIR Form Res. 2026 Sep 16;10:e87234. doi: 10.2196/87234 (PMC13628036; doi:10.2196/87234)
Supplement: Supplementary file 4 [file formative_v10i1e87234_app4.docx]

# **Multimedia Appendix 4. Interview Questions for Dementia Caregivers**

| Category | Question |
| --- | --- |
| Demographic questions | Can you provide brief information about yourself (age, gender, location, domicile, relationship with people with dementia, and how long you have been a caregiver)? |
| General Information for caregivers | How long has the person with dementia been diagnosed with dementia? |
|  | What is your main role in treating people with dementia (eg, monitoring health, helping with daily activities)? |
|  | Have you experienced any psychological problems since becoming a caregiver? If so, what challenges do you feel the most? |
| Challenges in treating people with dementia | What are the biggest challenges you face in treating people with dementia today? |
|  | In your opinion, are there any activities or processes that are most difficult to carry out when treating people with dementia? Why? |
|  | What strategies do you typically use to deal with behavior changes in people with dementia, such as confusion, aggression, or other unexpected behaviors? |
|  | Are there any specific challenges that you feel when monitoring the condition of people with dementia or managing their daily lives? |
| Security of personal data | Do you feel comfortable with the app storing the data of people with dementia, such as location or medication schedules? Why? |
|  | Why do you think it is important to ensure that data on people with dementia stay safe in this app? |
| Use of technology in caregiving | Have you ever used technology (such as apps or other tools) to support caregiving? If so, what app or technology do you use? |
|  | What types of features in the technology do you think are most helpful for supporting caregiving tasks? |
