# Supplementary material for: Caregiver Challenges and Functional Requirements for an mHealth App Supporting Home-Based Dementia Care: Qualitative Interview Study
Source: JMIR Form Res. 2026 Sep 16;10:e87234. doi: 10.2196/87234 (PMC13628036; doi:10.2196/87234)
Supplement: Supplementary file 5 [file formative_v10i1e87234_app5.docx]

**Multimedia Appendix 5.** Summary of challenges identified through content analysis.

| Challenges | Health workers | Caregivers | Nongovernmental organization employees |
| --- | --- | --- | --- |
| Lack of education and awareness regarding dementia symptoms and management | - “Many people with dementia already come in a state of moderate or severe dementia because from the beginning, the family is not aware of the symptoms that appear.” [Respondent NNI] - “Often, people with dementia experience disorders in executive functions, such as confusion and withdrawal from society. However, because they can still carry out normal activities, the family does not recognize this as an early sign of dementia.” [Respondent NNI] - “The main challenges in diagnosing dementia are low public awareness and the difficulty of early detection due to the lack of access to screening and experts.” [Respondent K] | - “Initially, we thought forgetting was natural for older people. But the longer it takes, the more significant the symptoms become. In one minute, you can repeat the same topic up to 3-4 times.” [Respondent IR] - “In the beginning, my in-laws often forgot. I think it’s common for older people. After all these years, I realized that this is not just ordinary senility.” [Respondent IR] - “At first, my in-laws were still able to work and do their usual activities but started to repeat the same questions often. We only realized after his behavior got out of control.” [Respondent AA] - “Five years ago, when Grandma was 76 years old, signs of dementia began to be seen. At first, it was just mild senility, but over time, the level increased.” [Respondent YDM] - “Opa was diagnosed only a year ago after being examined by a doctor, even though the symptoms have been happening for a long time.” [Respondent IJH] | - “The main challenge is the lack of understanding and awareness about dementia in the community, including among health workers. Many experts do not have enough knowledge in how to handle people with dementia optimally.” [Respondent ALZI] |
| Caregivers experiencing stress and mental strain while caring for people with dementia | - “Sometimes people with dementia can get angry, even verbally and physically abusive. If caregivers are not prepared, they can be stressed and overwhelmed.” [Respondent NNI] | - “I often experience psychological distress and emotional exhaustion. The biggest challenge is dealing with the very rapid mood swings of people with dementia.” [Respondent IR] - “I made time to go to a psychiatrist because I had hit a low point. I felt like there was no support; everything had changed for me. Finally, I experienced depression, even almost burnout.” [Respondent AA] - “I experienced severe stress when facing the decline in the conditions of people with dementia, especially in terms of cognition.” [Respondent RPN] - “At the beginning, interns would be surprised if they encounter older people who have tantrums or are often angry. Many were crying.” [Respondent LNY] | - “Many caregivers feel overwhelmed because they have to deal with people with dementia almost 24/7 without adequate support. Lack of rest time often has an impact on their physical and mental health.” [Respondent ALZI] - “People with dementia often experience extreme mood swings, such as irritability, irritability, or delusions. This means caregivers must have a good level of patience and communication strategies.” [Respondent ALZI] - “Caregivers are often overwhelmed by managing people with dementia, who constantly repeat questions or experience drastic emotional changes. In addition, physical and emotional fatigue is also a big challenge for caregivers.” [Respondent LNA] |
| Difficulties in monitoring the health and daily lives of people with dementia | - “The continuity of caregiving is a problem because many families do not have a permanent caregiver, causing exercise or therapy not to be carried out consistently.” [Respondent NNI] | - “Ensuring that the person with dementia takes medication regularly is very difficult because he often forgets. Sometimes, I have to remind him many times, but in a matter of minutes, he can forget again.” [Respondent IR] | - “mHealth that can remind [people of] medication schedules, provide video-based education, and allow remote consultations with experts will be very useful.” [Respondent ALZI] |
| Risk of getting lost and difficulty asking for help in an emergency among people with dementia | - “The use of wearable devices can also help monitor people with dementia’s activities in real time to prevent incidents such as getting lost or falling.” [Respondent K] | - “Previously, the condition of the person with dementia worsened, and he got lost, so I used the GPS [global positioning system] on my bike to monitor his location.” [Respondent RPN] - “Supervision must also be carried out almost all the time because there is a risk of people with dementia experiencing disorientation and getting lost if left alone.” [Respondent IR] | - “My husband, who has an IT background, is developing a panic button that can be used by older people if they need help. This tool is connected directly to the nurse’s mobile phone. However, we still face technical obstacles, such as unstable internet networks. The implementation of technology is urgently needed, especially for monitoring and rapid response to people with dementia conditions.” [Respondent LNA] |
| Barriers impeding access to health services | - “Many of my patients with dementia have to come all the way to the hospital for a short consultation. This requires travel and time, so it is not effective because access to services to hospitals in some areas is still difficult, or hospitals are far from where they live.” [Respondent H] | - “Families are using telemedicine to consult with doctors online, especially when they do not want to go to the hospital. This helps them get medical advice without having to wait a long time at the clinic.” [Respondent IR] | - “In Indonesia, mHealth are needed to bridge the limitations of access to health services, especially for families who live far from medical facilities.” [Respondent ALZI] |
| Declining cognitive function among people with dementia | - “Many people with dementia are not detected early because families only notice symptoms when people with dementia begin to experience significant memory loss or have difficulty carrying out daily activities.” [Respondent H] - “Symptoms that often occur are confusion, withdrawal, and loss of confidence. People with dementia also experience sleep disturbances, which then worsen their cognitive state.” [Respondent NNI] | - “The biggest difficulty in treating people with dementia is accepting the fact that [they] have worsening cognitive decline. In addition, the COVID-19 pandemic exacerbated the conditions of people with dementia, causing [them] to refuse to eat and be reluctant to carry out daily activities.” [Respondent RPN] | - “People with dementia often experience cognitive impairments, such as forgetting the closest people, confusion about time and place, and difficulty understanding conversations. This makes it difficult for them to carry out daily activities, such as dressing, eating, or managing finances.” [Respondent ALZI] |
