# Supplementary material for: Caregiver Challenges and Functional Requirements for an mHealth App Supporting Home-Based Dementia Care: Qualitative Interview Study
Source: JMIR Form Res. 2026 Sep 16;10:e87234. doi: 10.2196/87234 (PMC13628036; doi:10.2196/87234)
Supplement: Supplementary file 6 [file formative_v10i1e87234_app6.docx]

**Multimedia Appendix 6.** Mapping of challenges, features, principles of intervention theory, and health efforts.

| Challenges faced and features required | | Feature explanation | Intervention theory principles | Health efforts |
| --- | --- | --- | --- | --- |
| Lack of education and awareness regarding dementia symptoms and management (challenge faced): education with expert-validated content, providing information on the following:   ● Causes of dementia ● Symptoms of dementia ● Dementia treatment guide ● Dementia care ● Management of extreme behavioral changes in people with dementia ● Common diseases in older people ● Nutrition guide for older people ● Creating a friendly environment for people with dementia ● Early detection of dementia symptoms | | The educational feature provides expert-validated content about dementia, including its causes, symptoms, treatment, nutrition, early detection, and management of behavioral changes in people with dementia. | Valid and useful information | Promotive, because it provides caregivers with knowledge about dementia and care for older people, thereby increasing awareness and their ability to make care decisions. This is consistent with the definition of health promotion as a process that allows individuals to gain greater control over their health. |
| **Caregivers experiencing stress and mental distress while caring for people with dementia** | | | | |
|  | Assessment of psychological problems | This feature provides psychological tests or questionnaires that help caregivers and experts identify caregivers’ levels of stress, anxiety, or depression. | Valid and useful information | Preventive, because it allows caregivers to detect potential mental health disorders, such as stress and burnout, at an early stage. This is consistent with the principle of secondary prevention in health services, which aims to avoid worsening psychological conditions. |
|  | Caregiver community forum features (support group) | This feature allows caregivers to share experiences, receive emotional support, and exchange tips and strategies on caring for people with dementia through discussion forums or support groups. | Internal commitment | Promotive, because it provides a forum for social support and interaction between caregivers that strengthens emotional connections, enthusiasm, and coping capacity. This approach reflects community-based health promotion, which encourages empowerment and social solidarity. |
| **Difficulties in monitoring the health and daily lives of people with dementia** | | | | |
|  | Daily routine reminders for people with dementia (medication reminders and daily tasks) | This feature helps caregivers manage the daily schedules of people with dementia, covering taking medication, eating, and other important activities, with automatic notifications. | Internal commitment | Curative, because this feature supports the regularity of older people’s daily activities, such as taking medication and doing cognitive exercises, as part of the ongoing treatment process. The curative approach includes actions to address ongoing conditions and help maintain the stability of the patient’s condition. |
|  | Journaling for people with dementia | This feature allows caregivers to record their feelings, daily activities, and the development of the conditions of people with dementia. | Valid and useful information | Curative, because it systematically records the daily condition of older people to support medical decision-making by caregivers or professionals. Documentation of patient conditions is an important part of a curative approach in the context of monitoring and evaluating care. |
| **Barriers impeding access to health services** | | | | |
|  | Online consultation feature with health workers | This feature provides direct access to experts for online consultation regarding the conditions, treatment, and symptom management in people with dementia. | Valid and useful information | Curative, because it provides caregivers with direct access to consultations with physicians or psychologists to address ongoing health conditions or complaints. This intervention is part of a curative service that focuses on the diagnosis and treatment of diseases by professionals. Online consultation services like this also support the integration of primary health systems with technology to expand access to medical care. |
|  | Appointment submission feature for home care and basic treatment services | This feature allows caregivers to schedule expert visits to their homes to provide basic health services for people with dementia. | Internal commitment | Curative, as it facilitates face-to-face medical services needed to address ongoing health conditions, especially cases that cannot be resolved through online services. The curative approach includes access to professionals for direct medical diagnosis and therapy. |
| **Declining cognitive function among people with dementia** | | | | |
|  | Education in the form of videos and articles containing information about meaningful activities for people with dementia | This feature provides educational content that helps caregivers and people with dementia find activities that can improve their mental and physical well-being. | Valid and useful information | Promotive, because it provides caregivers with knowledge about dementia and care for older people, thereby increasing awareness and their ability to make care decisions. It fits the definition of health promotion as a process that allows individuals to gain greater control over their health. |
|  | Music playlist features | This feature provides curated music collections to soothe, evoke memories, or stimulate positive emotions in people with dementia. | Free informed choice | Rehabilitative, because it provides familiar music that can awaken memories and positive emotions in older people. Music therapy has been shown to be effective as part of a rehabilitative approach, especially for patients with neurocognitive impairment. |
|  | Games and quizzes | The games and quizzes feature provides simple games and quizzes with interactive displays and clear visual instructions to exercise the memory and attention of the people with dementia in a fun and accessible manner. | Free informed choice | Rehabilitative, because this feature trains memory and concentration of older people through a fun approach based on interactive games. These activities are included in nonpharmacological cognitive therapies that support the restoration of brain function. |
